# High-performance perovskite CH_3_NH_3_PbI_3_ thin films for solar cells prepared by single-source physical vapour deposition

**DOI:** 10.1038/srep29910

**Published:** 2016-07-18

**Authors:** Ping Fan, Di Gu, Guang-Xing Liang, Jing-Ting Luo, Ju-Long Chen, Zhuang-Hao Zheng, Dong-Ping Zhang

**Affiliations:** 1 Institute of Thin Film Physics and Applications, College of Physics and Energy, Shenzhen University, 518060, China

## Abstract

In this work, an alternative route to fabricating high-quality CH_3_NH_3_PbI_3_ thin films is proposed. Single-source physical vapour deposition (SSPVD) without a post-heat-treating process was used to prepare CH_3_NH_3_PbI_3_ thin films at room temperature. This new process enabled complete surface coverage and moisture stability in a non-vacuum solution. Moreover, the challenges of simultaneously controlling evaporation processes of the organic and inorganic sources via dual-source vapour evaporation and the heating process required to obtain high crystallization were avoided. Excellent composition with stoichiometry transferred from the powder material, a high level of tetragonal phase-purity, full surface coverage, well-defined grain structure, high crystallization and reproducibility were obtained. A PCE of approximately 10.90% was obtained with a device based on SSPVD CH_3_NH_3_PbI_3_. These initial results suggest that SSPVD is a promising method to significantly optimize perovskite CH_3_NH_3_PbI_3_ solar cell efficiency.

Organic-inorganic hybrid perovskite based on methylammonium lead halide (MAPbX_3_, where MA is an organic cation (CH_3_NH_3_^+^), Pb is a divalent metal ion (Pb^2+^) and X is a halide (I^−^ or Cl^−^))^1^ is currently being widely studied owing to its variety of interesting optical, magnetic and electronic properties^2^,^3^,^4^. Since perovskite solar cells (PSCs) were first introduced by the Miyasaka group^5^ in 2009, MAPbX_3_ has emerged as the forerunner in the next-generation of photovoltaic technology. To date, within just 6 years, the power conversion efficiency (PCE) of perovskite solar cells has most recently reached 20.8%^6^. The achieved success is closely associated with the specific photoelectrical properties of perovskite light absorbers, such as the optimal and direct band gap, high absorption coefficient, charge transport property, and long-term charge life^7^,^8^,^9^,^10^,^11^.

Along with advantages of organic-inorganic hybrid perovskites, various methods have been used to prepare MAPbI_3_ thin films. These methods include the one-step spin-coating method^12^,^13^,^14^,^15^,^16^, the two-step sequential method^17^,^18^,^19^, the vapour-assisted solution process^20^, and the dual-source vapour evaporation method^21^,^22^,^23^,^24^. The one-step spin-coating method was employed to directly deposit the perovskite material from a precursor solution mixture of PbI_2_ and CH_3_NH_3_I in a polar solvent such as r-butyrolactone (GBL) or N,N-dimethyformide (DMF), followed by annealing at 70–150 °C to remove the additives and crystallize the perovskite thin films^14^,^16^. However, it is difficult to find a suitable solvent that can dissolve both components, and it is generally difficult to control the reaction rate between CH_3_NH_3_I and PbI_2_. To create a uniform perovskite film, the two-step sequential method has been developed. First, nano-structured TiO_2_ scaffolds are infiltrated by a highly concentrated PbI_2_ solution, and then PbI_2_ thin films are reacted with the CH_3_NH_3_I solution by dipping followed by annealing^17^,^25^,^26^,^27^. In general, the two-step sequential method offers better control over the perovskite morphology than the one-step spin-coating method. Unfortunately, the two-step sequential method leads to the dissolution of the perovskite film into the solution. As a variation to this method, a vapour-assisted solution process was demonstrated whereby the solution-processed PbI_2_ film is annealed onto compact TiO_2_ exposed to an MAI vapour; this method was used to fabricate efficient PV devices^20^. However, both the two-step sequential method and the vapour-assisted solution process make it difficult for CH_3_NH_3_I to access and react with the inner PbI_2_ layer, which leads to only the surface of the inorganic crystal layer being converted to the perovskite^26^,^28^. Thus, formation of a uniform, smooth, and continuous perovskite film via solution processes is challenging. By contrast, vapour deposition is a promising technique used in the thin-film solar cell industry (e.g., α-Si, Cu(InGa)Se_2_ and CdTe). Dual-source vapour evaporation was used to deposit a uniform thin film layer of MAPbI_3-x_Cl_x_ without pinholes and with complete surface coverage, and the co-evaporation of two precursors (PbCl_2_ and CH_3_NH_3_I) resulted in a solar cell with a PCE of 15.4%^21^. A distinct advantage of this technique over the solution-processing method is the enhanced control of film quality, thickness and morphology. However, it is difficult to balance the rate of the organic and inorganic sources and to control the reaction between the different vapour molecules of MAI and PbCl_2_ simultaneously, which easily leads to the presence of PbI_2_ impurities^21^,^23^.

Here, we demonstrate a facile and efficient method for the fabrication of perovskite thin films. First, high-quality single-crystalline MAPbI_3_ was prepared^10^,^29^,^30^. Then, using a single-source physical vapour-deposition (SSPVD) method, the MAPbI_3_ powder is converted to the gas phase by evaporation. Finally, the gas phase is transported and deposited back on the surface of the sample. This method is different from previous methods such as the one-step spin-coating method, two-step sequential method, vapour-assisted solution process, and dual-source vapour evaporation that have been used to prepare MAPbI_3_ thin films involving reactions between the organic and inorganic sources. Furthermore, the conventional methods require an invariable subsequent thermal annealing treatment to remove the additives and/or to crystallize perovskite thin films^31^,^32^. However, an inappropriate annealing time (excessive or inadequate) or temperature (too high or too low) could result in poor film morphology with pinholes or undesirable crystallization due to the difficulties in controlling the evaporation of solvents or crystallization of the perovskite thin film. By contrast, with the SSPVD method, MAPbI_3_ gases are guided to the sample and do not undergo a chemical reaction on the way to the sample or on the surface of the sample. Moreover, this method effectively avoids the problems such as the high reaction rate between CH_3_NH_3_I and PbI_2_, lack of suitable solvent for CH_3_NH_3_I and PbI_2_, impurities in the precursor solution, dissolution of the perovskite film, need for the simultaneous control of evaporation rates of the organic and inorganic sources, presence of PbI_2_ impurities, improper heat treatment, and so on. This method produces uniform, smooth, nonporous perovskite thin films with complete surface coverage, a high level of phase purity and good crystallization.

## Results

**Figure 1:**
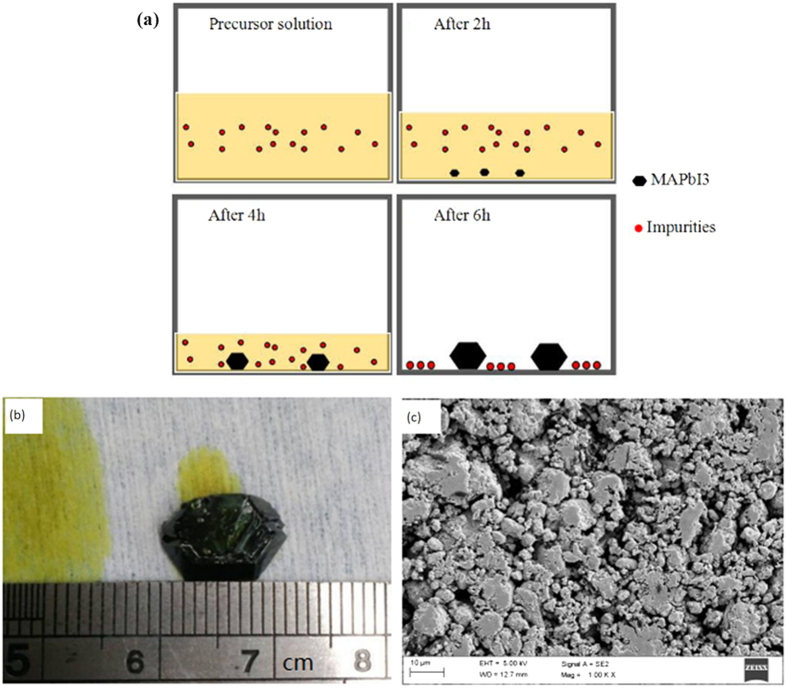
Progress of the MAPbI_3_ crystal growth (**a**), photos of MAPbI_3_ crystal (**b**) and MAPbI_3_ powder (**c**).

**Figure 2:**
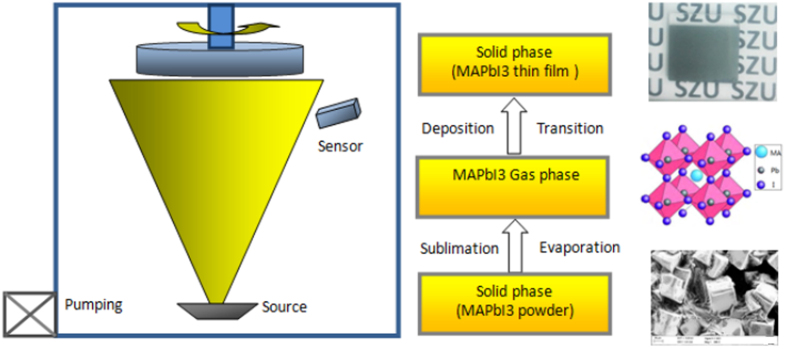
Single-source physical vapour-deposition process of the perovskite MAPbI_3_ thin film.

As shown in Fig. 1(a), the MAPbI_3_ crystal was grown by maintaining the precursor solution at 90 °C on the hot plate so that a high-quality MAPbI_3_ crystal with fewer impurities was obtained after some hours (Fig. 1(b)). The raw MAPbI_3_ crystal with fewer impurities is favourable for the prepared MAPbI_3_ thin film and solar cells. To easily evaporate MAPbI_3_, the MAPbI_3_ crystals were ground to small crystals or powder before use (Fig. 1(c)). As shown in Fig. 2, with the single-source physical vapour-deposition (SSPVD) method, the work current of the crucible was rapidly increased from 0 A to 100 A, and simultaneously, the temperature of the source was rapidly raised to the point at which the MAPbI_3_ powder either evaporates or sublimates efficiently without a chemical reaction; the MAPbI_3_ powder is then converted to the MAPbI_3_ gas phase by evaporation and the film is deposited on the substrates atomistically. The entire deposition process, conversion to the gas phase, and movement and condensation to the substrate, was such that only the physical morphology of the aggregate state of the material changes from solid to gaseous and back to solid. However, chemically, the MAPbI_3_ material remains the same. With this SSPVD method, MAPbI_3_ gases are guided to the substrate without undergoing a chemical reaction on the way to the substrate. Then, nucleation and crystallization of the MAPbI_3_ thin film on the substrate was facilitated by the mere effect of the removal of the gas phase of MAPbI_3_. Therefore, the use of subsequent thermal annealing treatment to evaporate the solvent or crystallize the perovskite film is unnecessary. Owing to the advantages of the single-source physical vapour-deposition process without reaction, this method is a facile and attractive approach for the fabrication of high-quality perovskite films.

**Figure 3:**
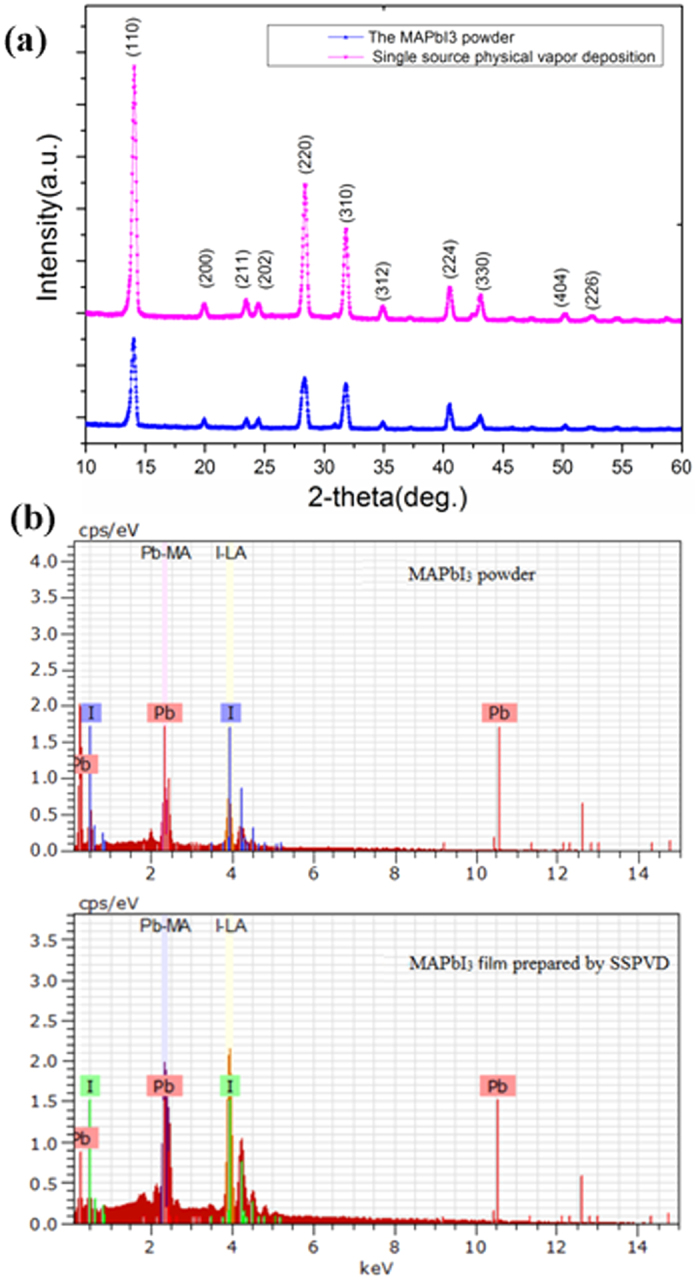
XRD patterns of MAPbI_3_ powder and film prepared by SSPVD (**a**), EDS spectral line pattern of MAPbI_3_ powder and film prepared by SSPVD (**b**).

X-ray diffraction (XRD) measurements were used to further examine the formation of perovskite phases and crystallization. As shown in Fig. 3(a), a set of preferred orientations at 14.08°, 28.44°, 31.85°, 40.58° and 43.19° was observed, with these assigned to the (110), (220), (310), (224) and (330) planes of the MAPbI_3_ perovskite tetragonal structure^33^, respectively. Minor peaks of the (200), (211), (202), (312), (404) and (226) planes are present at 2θ values of 19.92°, 23.54°, 24.52°, 34.94°, 50.22°, and 52.54°, respectively, clearly indicating that all perovskite films or powder are of high phase purity. Furthermore, there are no MAI or PbI_2_ phase peaks in the powder or the film, confirming that the samples are also compositionally pure. Notably, the spectrum of the single-source physical vapour-deposited perovskite film is similar to the spectrum of the source of the perovskite powder, confirming that the film and powder consist of the same MAPbI_3_ material. Furthermore, owing to the change of the material in physical morphology from the MAPbI_3_ crystal to the MAPbI_3_ thin film, the perovskite film peaks became more intense. All analyses clearly indicate that the conversion of the MAPbI_3_ crystal to the MAPbI_3_ thin film only changes the physical morphology during the single-source physical vapour-deposition process, without undergoing a chemical decomposition. These findings are further supported by the EDS spectral line pattern and elemental composition.

**Figure 4:**
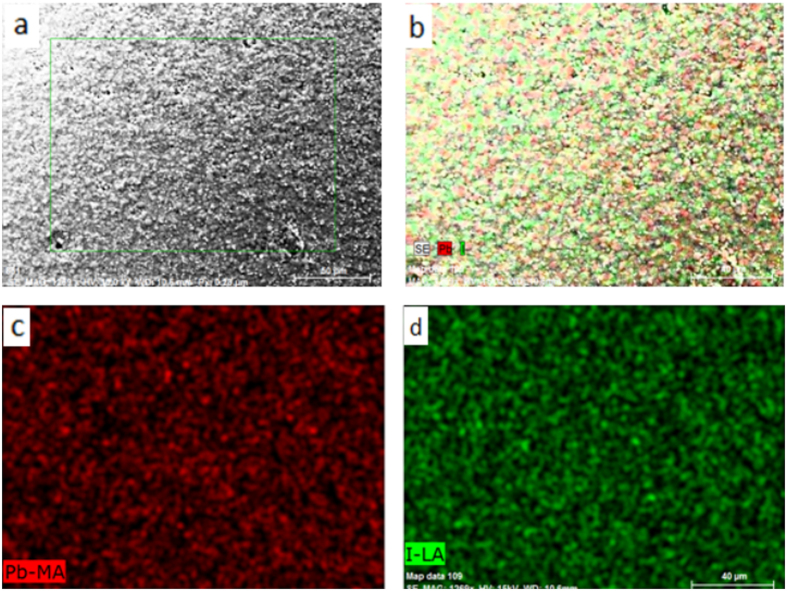
SEM-BSD morphologies (**a**) and EDS-Mapping of the MAPbI_3_ film (the combination map of the Pb and I (**b**), Pb (**c**) and I (**d**).

The EDS technique was used to analyse the composition and element ratio of the MAPbI_3_ powder and the MAPbI_3_ film prepared by SSPVD. As shown in Fig. 3(b), there are two feature peaks at 2.48 and 3.98 keV, which are assigned to the Pb and I elements, respectively. The EDS spectral line pattern of the perovskite film is similar to the spectrum of the source perovskite powder, clearly indicating their homogeneity. The similar value of approximately 0.339 and 0.334 are obtained for the Pb/I ratio of the film grown via single-source physical vapour-deposition and for the MAPbI_3_ powder, respectively. Both values are close to the theoretical stoichiometry value of 0.333, confirming that the perovskite film prepared by SSPVD has the same composition as the MAPbI_3_ powder. These findings are supported by the XRD analysis results discussed above. Furthermore, examination of the EDS-Mapping presented in Fig. 4 shows that Pb and I are well distributed on a large scale, confirming the uniformity of the MAPbI_3_ film grown via the SSPVD method.

**Figure 5:**
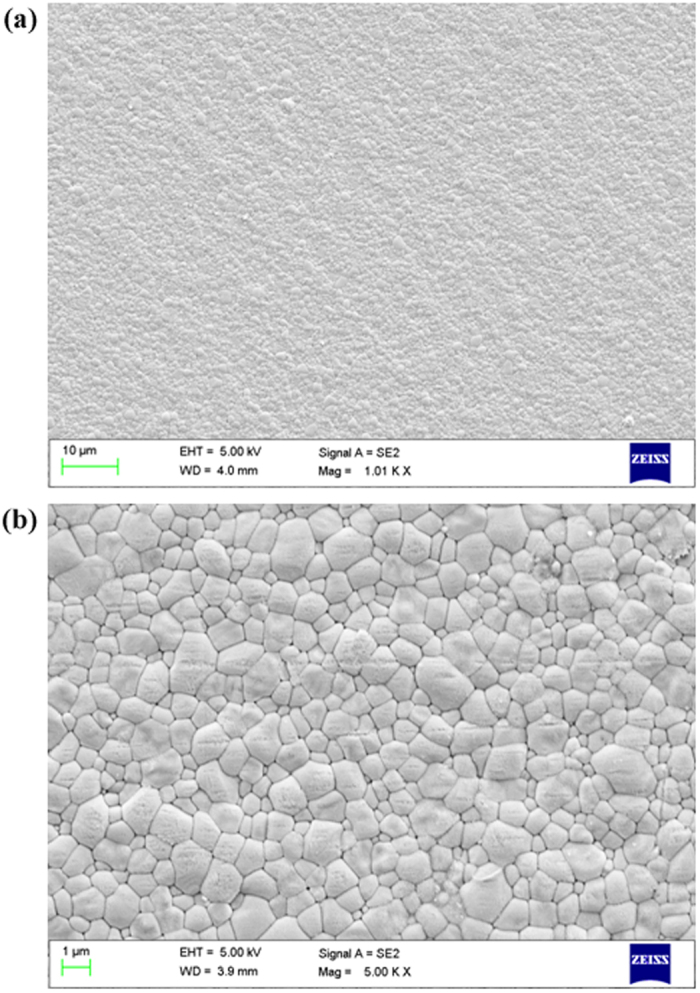
SEM images of MAPbI_3_ thin films (low-magnification SEM image (**a**) and high-magnification SEM image (**b**)).

As can be clearly observed in Fig. 5, the films prepared by single-source physical vapour-deposition are extremely dense, compact and uniform even on a large scale as shown in the low-magnification SEM image (Fig. 5(a)), indicating that this method will be suitable for fabricating high-quality perovskite solar cells over a large area. At the same time, the high-resolution SEM image (Fig. 5(b)) reveals that the as-deposited perovskite films without the subsequent thermal annealing treatment also possess the characteristics of full surface coverage on the substrates without pinholes but with remarkable grain size of up to 2 μm, indicating a higher level of crystallization compared with the dual-source vapour-evaporation processed films. The uniform, smooth, nonporous and complete surface coverage of the perovskite thin film without heat-treating suggests that these films are promising for applications in flexible solar cells. These results confirm the progress of the nucleation and crystallization of MAPbI_3_ from the gas phase to the thin film without the need of subsequent thermal annealing treatment. These impressive characteristics could be due to the ability to easily control the evaporation rate, time, and pressure with high reproducibility in the single-source physical vapour-deposition process without reaction.

**Figure 6:**
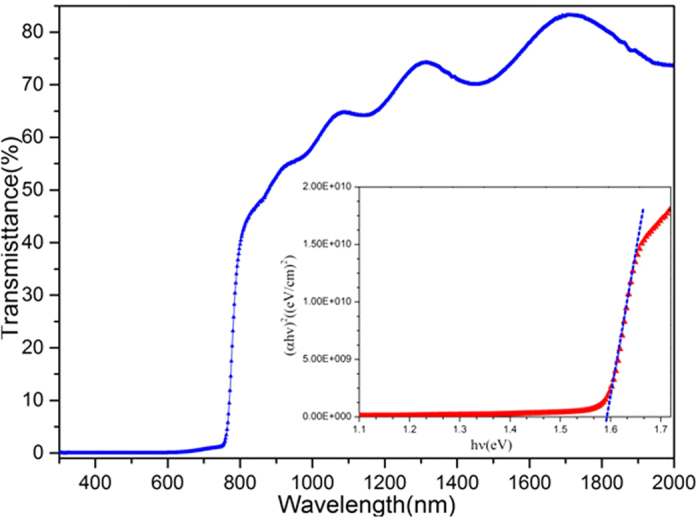
Transmitted spectrum and the optical band gap estimation (insert) of MAPbI_3_ thin film.

Figure 6 shows the transmittance spectra as a function of wavelength for the MAPbI_3_ film prepared by the single-source physical vapour-deposition method. The transmittance of the MAPbI_3_ film changed from 80% to 49% slowly in the wavelength span from 2000 nm to 800 nm. However, the transmittance decreased drastically at 800 nm, confirming the existence of a sharp absorption edge^11^. Last, the transmittance was low and close to 0% in the wavelength range from 765 nm to 350 nm, indicating that good crystallinity of the MAPbI_3_ films was obtained, making this material a good light-absorber over the entire visible solar emission spectrum. Additionally, the band gap energy was calculated according to


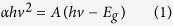


where *α* is the absorption coefficient, *hv* is the photon energy, *A* is a constant, and *E* is the band gap energy. As shown in Fig. 6, the results revealed that the band gap of the MAPbI_3_ thin film is approximately 1.59 eV, which is close to the theoretical value of 1.55 eV reported by Baikie^11^.

**Figure 7:**
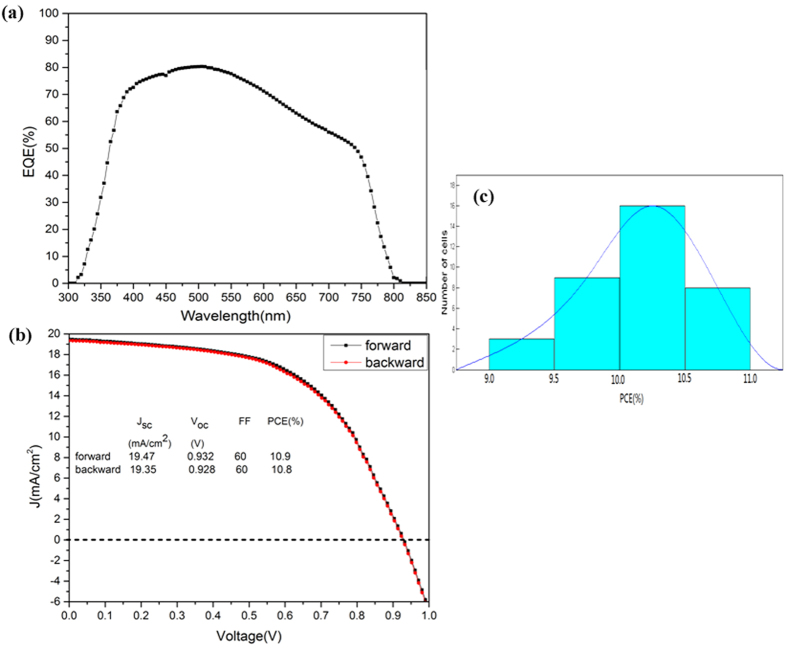
External quantum efficiency spectra for the best perovskite solar cell (**a**), *J-V* characteristic curve (**b**) and PCE histograms of MAPbI_3_ thin film solar cells (**c**).

Figure 7(a) shows that the EQE rises at the same wavelength, reaching a maximum value of 80.1% in the 475–525 nm wavelength range. The current density-voltage (*J-V*) characteristics of the solar cell based on the best performing device reported in Fig. 7(b) exhibited a *J*_*SC*_ of 19.47 mA/cm^2^, a *FF* of 60%, and a *V*_*OC*_ of 0.932 V, leading to a high PCE of 10.9%. The *J-V* curve is almost independent of the scan direction, indicating that no obvious photocurrent hysteresis occurred. The observed small reduction may be due to the high humidity present in the test environment (approximately 60–78%). The statistics for the PCE based on 36 devices are shown in Fig. 7(c). The figure shows that a narrow distribution of PCE values in the 9.0–11.0% range with the average value of 10.2 ± 0.1% was obtained. The improved reproducibility in device performance is closely related to the reproducibility of MAPbI_3_ films prepared by SSPVD.

## Conclusion

In summary, we report SSPVD, a facile, efficient and reproducible method for the fabrication of perovskite thin films. This method involves the process of converting the MAPbI_3_ powder to the gas phase, transferring the MAPbI_3_ gas to the samples and then condensing the MAPbI_3_ gas onto the surface of the substrates.During this process only the physical morphology of the aggregate state of the material changes, which is different from the methods reported that are based on the reaction process between the organic and inorganic sources. A perovskite thin film with uniform, smooth, nonporous and complete surface coverage as well as with a high level of phase purity and good crystallization was formed via SSPVD. The initial results for the device show that a PCE of approximately 10.90% was obtained for MAPbI_3_ grown by SSPVD, suggesting that this method is promising for further significant optimization and application in larger surface area perovskite MAPbI_3_ solar cells with higher PCEs.

## Methods

### Material preparation

CH_3_NH_3_I synthesis: first, methylammonium iodide (MAI) was synthesized by reacting hydroiodic acid (HI) (60 ml, 57 wt% in water, Sigma Aldrich) and methylamine (CH_3_NH_2_) (56 ml, 40 wt% in water, Sigma Aldrich) in a 250 ml round bottom flask at 0 °C for 2 h with constant magnetic stirring. Second, the white methylammonium iodide powder was crystallized by evaporating solvents at 90 °C for 2 h. Then, the crystallized white methylammonium iodide powder was purified with diethyl ether three times. Subsequently, methylammonium iodide was dried at 60 °C in vacuum overnight. Finally, the white methylammonium iodide powder was saved at 25 °C in a vacuum oven and was desiccated before use.

Preparation of the precursor solution and MAPbI_3_ crystal: To generate the perovskite solution, CH_3_NH_3_I (0.8 g) and PbI_2_ (2.3 g, 99.99%, Sigma Aldrich) were dissolved in r-butyrolactone (100 ml, 99%, Sigma Aldrich) in a 250 ml MAPbI_3_ round bottom flask at 60 °C for 10 h with constant magnetic stirring. Then, the MAPbI_3_ precursor solution was maintained at 90 °C on the hot plate. After 2 h, there were many small seed crystals grown with solvent evaporating as the same time. Then, the two largest seed crystals were kept to grow, and others were discarded. The above step was repeated until all precursor solution was evaporated. Finally, the MAPbI_3_ crystals were ground to powder before use.

### Film preparation

Single-source physical vapour-deposition method: K9 glass substrates with the thickness of approximately 1 mm were ultrasonically cleaned in acetone, alcohol and deionized water, sequentially. Then, 0.6 g of MAPbI_3_ powder was placed into a crucible. When the pressure in the chamber was pumped down to below 1 × 10^−3 ^Pa, the work current of the crucible was rapidly increased from 0 A to 100 A, and at the same time, the temperature of source was raised rapidly to the point at which the MAPbI_3_ powder either evaporates or sublimates efficiently. The value of the deposition rate of the thin film on the sensor was approximately 8.5 ± 1.3 Å/s. There is a close relationship between the power and the rate of deposition. The total process of the deposition was approximately 3 min and then the sample shutter was closed. The value of the thickness of the thin film on the sensor was approximately 8.9 kÅ. As the same time, the substrate holder was rotated at a rate of 5 rpm to ensure uniform evaporation. The actual deposited equivalent thickness was approximately 400 nm.

### Device fabrication

ITO-coated glass substrates (10 Ohm/sq, YINKOU OPVTECH) were cleaned with ethyl alcohol and deionized water in a sonicator for 15 min each. Then, the cleaned substrates were treated by oxygen plasma for 30 min. A thin layer of PEDOT:PSS (CLEVIOS PVP AI4083,80 nm) was fabricated by spin-coating, followed by annealing at 120 °C on a hot plate for 15 min. A perovskite layer was deposited on top of the PEDOT:PSS layer via the SSPVD method. A PCBM solution (10 mg in 1 ml chlorobenzene, 100 nm) was spun on the prepared perovskite film. Finally, 80 nm thick Ag electrodes were deposited using a thermal evaporator. The vapour pressure was 1 × 10^−3 ^Pa inside the vacuum chamber and the value of the Ag deposition rate on the sensor was approximately 12 Å/s.

### Characterization

#### SEM

The perovskite film morphology was analysed using a SUPRA 55 scanning electron microscope (SEM) using an electron beam accelerated at 5 kV.

#### EDS

The composition and element mapping of films and perovskite powder were obtained using an energy dispersive X-ray microanalysis system (Model: BRUKER QUANTAX 200) attached to the SEM.

#### XRD

Crystallographic structures of films were analysed by X-Ray diffraction (XRD) technique (Ultima IV) with CuKα radiation (0.15406 nm) operated at 40 kV and 40 mA using 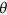/2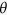 scans, whereas perovskite powder was analysed using 2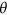 scans.

#### Optical transmittance

Optical transmittance properties of the perovskite films were obtained using an ultraviolet (UV)/visible (VIS)/near-infrared (NIR) spectrophotometer (Lambda 950, Perkin Elmer).

#### Film thickness

The prepared film thickness was measured using a DEKTAK XT profilometer.

#### Devices measurement

The solar cells were tested in air, the temperature was approximately 27 °C, and the humidity was between 65% and 75%. The current density-voltage (J-V) characteristics of the perovskite cells were measured under simulated AM 1.5G conditions (100 mW/cm^2^) with a Keithley 2400. The voltage was scanned from 0 V(1 V) to 1 V(0 V) with the scan rate of approximately 0.1 V/s. The active area was approximately 9 mm^2^. The EQE spectra were acquired under 1.5 AM white light using an EQE 200 Oriel integrated system. The measurement step was 10 nm and the photocurrent was recorded using a lock-in amplifier.

## Additional Information

**How to cite this article**: Fan, P. *et al*. High-performance perovskite CH_3_NH_3_PbI_3_ thin films for solar cells prepared by single-source physical vapour deposition. *Sci. Rep*. **6**, 29910; doi: 10.1038/srep29910 (2016).
